# Green Manure Crops as Food Source: Impact on the Performance of the Migratory Beet Webworm, *Loxostege sticticalis* (Lepidoptera: Pyralidae)

**DOI:** 10.3390/insects14080693

**Published:** 2023-08-05

**Authors:** Lin Ma, Yaolu Tang, Lei Zhang, Xingfu Jiang

**Affiliations:** State Key Laboratory for Biology of Plant Diseases and Insect Pests, Institute of Plant Protection, Chinese Academy of Agricultural Science, Beijing 100193, China; linma1990@163.com (L.M.); tangyaolu309@163.com (Y.T.); leizhang@ippcaas.cn (L.Z.)

**Keywords:** *Loxostege sticticalis*, green manure crops, host fitness, life history, flight ability

## Abstract

**Simple Summary:**

Green manure application is an effective management practice for enhancing soil nutrient levels and organic matter, but pest management in green manure fields has traditionally been ignored. The beet webworm, a polyphagous insect, is currently experiencing an outbreak in northern China, and represents a significant migratory pest. We conducted a comprehensive evaluation of the effects of green manure crops on beet webworm by measuring its growth, development, fecundity, and flight ability on *Chenopodium album* (a suitable host), as well as three major legume green manure crops in China: *Pisum sativam*, *Vicia sativa*, and *Vicia villosa*. Our findings indicate that *V. villosa* does not serve as a host plant for the beet webworm, which experiences 100% mortality during its larval stage. This indicates that the large-scale cultivation of *V. villosa* as a green manure crop in northern China will not increase the risk of the beet webworm outbreaks. Although the beet webworm has a significantly lower host adaptability to *P. sativam* and *V. sativa* compared to *C. album*, it is still well adapted to these two green manure crops. Our research provides a foundation for selecting appropriate green manure varieties and implementing effective pest control measures during their cultivation.

**Abstract:**

The application of green manure is crucial for achieving sustainable agriculture and animal husbandry, but pest management is often overlooked. Conducting a risk assessment for insect pests in green manure is essential. The beet webworm, *Loxostege sticticalis*, a polyphagous insect, is currently experiencing an outbreak in northern China, and represents a significant migratory pest. A two-sex life table and flight mill test approach was used to comprehensively evaluate the effects of three major legume green manure crops (*Pisum sativam*, *Vicia sativa*, and *Vicia villosa*) on the growth, development, fecundity, and flight ability of *L. sticticalis* in China. Our findings indicate that *L. sticticalis* cannot utilize *V. villosa* for generational development. *L. sticticalis* shows reduced performance on *P. sativam* and *V. sativa* compared to its suitable host *Chenopodium album*. However, both the population parameters (*R_0_*, *r*, *λ*, and *T*) and the population prediction results suggest that *L. sticticalis* can adapt to *P. sativam* and *V. sativa*. In the process of promoting green manure, careful consideration should be given to the selection of appropriate green manure varieties and the implementation of effective pest control measures during their planting. Our findings lay the groundwork for the promotion of green manure and implementation of an ecological management plan for *L. sticticalis*.

## 1. Introduction

The green manure industry plays a crucial role in ensuring and promoting the sustainable development of agriculture and animal husbandry in China [1,2,3]. On the one hand, green manure application is an effective management practice and method for enhancing soil nutrients and improving the structure microbial communities, ultimately leading to increased crop quality and yield [2,4,5,6,7]. In addition, some species of green manure crops can also be used as high-quality forage grass for animal husbandry [8,9,10]. In recent years, China has been actively promoting green ecology by continuously improving the technical system for efficient production of green manure in agricultural areas and orchards, as well as enhancing fertilizer saving, efficiency, and quality [1,11]. Additionally, China has proposed an industrial mechanism called “green manure +” to promote ecological restoration technology and industrial application based on green manure. Green-manure-based rotations have been shown to be an effective sustainable agriculture practice [12,13,14,15]. However, green manure crops that are planted can be exploited by pests, which can prolong the occurrence duration and number of pests, resulting in unexpected pest outbreaks [16]. Moreover, most growers have traditionally ignored pest management in green manure fields. Therefore, it is important to carry out pest risk assessment based on green manure.

The beet webworm, *Loxostege sticticalis* L. (Lepidoptera: Pyraloidae: Crambidae), is a global pest, found in a wide belt zone between 36°N and 55°N, with periodic outbreaks occurring in many countries across Asia, Europe, and North America [17,18]. *Loxostege sticticalis* is a significant migratory pest in agriculture and animal husbandry across North, Northeast, and Northwest China [17,19]. *Loxostege sticticalis* can cause damage to over 259 species of host plants belonging to 48 families [20]. In the year of its major occurrence, the local crop yield loss in China could reach 60%, even leading to crop failure, which poses a significant threat to the security of agriculture and animal husbandry production for grain, oil, and forage crops [19]. In 2023, the Ministry of Agriculture and Rural Affairs of the People’s Republic of China issued the “List of Class I Crop Pests and Diseases”, which ranked *L. sticticalis* as the third most damaging pest among ten (http://www.moa.gov.cn/govpublic/ZZYGLS/202303/t20230314_6422981.htm, access on 7 March 2023). Periodic outbreaks are a characteristic of *L. sticticalis* occurrence [21], with three such occurrences in China since 1949: from 1952 to 1960, from 1977 to 1986, and from 1995 to 2010 [22,23,24]. Currently, China is experiencing its fourth outbreak cycle of *L. sticticalis* [25].

There are abundant green manure germplasm resources in China [26], and various pests that attack green manure crops have been identified, including moths, aphids, thrips, leaf bugs, and others [27]. Migratory pests, such as armyworms, often feed on the nectar of legume green manure crops such as *Astragalus sinicus* L. and *Vicia sativa* L. to obtain nutrients and energy during long-distance migration [28]. However, the risks and impacts of pests on green manure crops are still poorly understood [29,30], and even less information is available regarding the effects of *L. sticticalis* on green manure crops. As green manure crops become more popular, it is important to determine whether they will be harmed by infestations of this pest in order to provide technical support for its scientific prevention and control as well as promote healthy development within the industry.

As mentioned above, there is a relative lack of research on green manure pests, while *L. sticticalis* is currently in an outbreak cycle in China. For those reasons, we have chosen a suitable host (*Chenopodium album* L.) for *L. sticticalis*, and three legume green manure crops (*Pisum sativum* L., *Vicia sativa* L., and *Vicia villosa* Roth.), which have been widely promoted and applied in the ‘Three North’ regions of China [2]. Through establishing two-sex life tables for *L. sticticalis* feeding on different host plants and conducting adult flying tests in doors, we aim to clarify the impact of various green manure crops on the growth, survival, reproduction, and flight ability of *L. sticticalis*. This will provide experimental data support for improving control measures against this migratory pest and promoting the application of green manure in China.

## 2. Materials and Methods

### 2.1. Laboratory Colony of Insects

A laboratory colony of *L. sticticalis* was established by collecting diapausing pupa from the suburbs of Kangbao (114.45°E, 41.73°N), Hebei province, in China. This colony was fed on common lambsquarters, *Chenopodium album* L., for three generations before this experiment. Larvae were reared under controlled conditions of 22 ± 1 °C, 70% ± 5% relative humidity (RH), and a photoperiod of 16:8 h (L:D). When larvae of *L. sticticalis* reach maturity and cease feeding, it is recommended to provide a layer of sterilized sandy loam that is 7–10 cm thick with a water content of approximately 20%. This will prepare the larvae for burrowing into the soil to create cocoons and pupate until they emerge as adults. The adults were given a 10% glucose–water solution (*w*/*v*) as a nutritional supplement. The colony is sustained by multiple pairs of male and female adults mating with each other, while sulfuric acid paper is provided for the females to deposit their eggs after copulation. The egg masses are collected daily, labeled with the date and placed in glass jars.

### 2.2. Green Manure Crops and Growth Conditions

In this study, *Chenopodium album* L. (a suitable host plant for *L. sticticalis*) and three green manure crops (the pea (*Pisum sativum* L.), the common vetch (*Vicia sativa* L.), and the hairy vetch (*Vicia villosa* Roth.)) were planted to serve as feeding host plants for the larvae of *L. sticticalis.* All host plants were cultivated in a greenhouse (22 ± 1 °C) located at the Institute of Plant Protection, Chinese Academy of Agricultural Sciences, Beijing, China. The water and fertilizer conditions were consistent for all host plants, and no pesticides were used during planting. When the plants reached a height of 30–40 cm (approximately 20 days old), the leaves and stems from the top 15–20 cm were removed and utilized as food for larvae of *L. sticticalis*.

### 2.3. Life History Characteristics of Loxostege Sticticalis Feeding on Different Green Manure Crops

Two-sex life tables were constructed as described by Chi et al. [31,32] to assess the development, survival, and reproduction of *L. sticticalis* fed on *C. album* and three different green manure crops (*P. sativum*, *V. sativa*, and *V. villosa*), which were divided into four groups based on their respective feeding host plants. For each group, 120 eggs from the same batch were placed in 750 mL glass jars and provided with fresh host plants corresponding to their group. Egg hatching was observed and recorded daily. Newly hatched larvae were each placed into a 45 mL clear, finger-shaped glass tube and labeled with numbers. Adequate fresh host plants were provided and replaced daily, while the survival, developmental stage, and duration of each larva were observed and recorded every day. When the larvae had completed their fifth instar and ceased feeding, they were individually transferred to finger-shaped tubes containing approximately 3 cm of sterilized sandy loam with a water content of around 20%. The pupal stage was then observed and recorded. The newly emerged females were individually paired with young males from the same group in a glass tube (5 × 12 cm diameter × height) and covered with cotton gauze as the oviposition substrate. The pairs were fed 10% (*w*/*v*) glucose in sterile water. The survival and number of eggs laid were recorded daily until all *L. sticticalis* adults had died.

### 2.4. Pupal Weight of Loxostege Sticticalis Feeding on Different Green Manure crops

In order to avoid human interference affecting the accuracy of life table parameters, we determined the pupal weight of *L. sticticalis* feeding on different host plants separately and divided them into four groups based on their respective hosts. For each group, 120 eggs from the same batch were placed in 750 mL glass jars and provided with fresh host plants corresponding to their group. Newly hatched larvae were each placed into a 45 mL clear, finger-shaped glass tube. Adequate fresh host plants were provided and replaced daily. When the larvae had completed their fifth instar and ceased feeding, they were individually transferred to finger-shaped tubes containing approximately 3 cm of sterilized sandy loam with a water content of around 20%. Pupation was observed every day. On the seventh day of pupation (counting from drilling into the soil), the earth cocoon was sliced open, and the weight of each pupa was measured. Thirty pupae were randomly selected from each group for weighing.

### 2.5. Flight Ability of Loxostege Sticticalis after Feeding on Different Green Manure Crops

As described in previous studies [33], flight ability tests were conducted on adult *L. sticticalis* after feeding on different host plants using a 48-channel computer-interfaced flight mill system that automatically records parameters such as total flight duration, distance, and average velocity. Considering that 1–2-day-old adults exhibit lower flight activity [34], 3-day-old unmated females and males of *L. sticticalis* were selected for testing after feeding on different host plants. For each host group, 24 females and 24 males underwent the tests. Tested moths were anesthetized with ether, and scales at the dorsal junction of the thorax and abdomen were gently swept away. Short plastic tethers were glued to the cuticle with 502 adhesive glues (Beijing Chemical Co, Beijing, China). A tethered moth was attached to the arm of a flight mill. The tests were performed in darkness, at 22 ± 1 °C and 70 ± 5% RH, conditions promoting the maximum flight capacity of *L. sticticalis* [34]. All flight tests began at 19:00 h and continued for 12 h.

### 2.6. Data Analysis

The raw life table data were analyzed and the life history characteristics were estimated based on an age-stage, two-sex life table using TWOSEX-MSChart software (Version 2023, Laboratory of Theoretical Ecology, National Chung Hsing University, Taichung, Taiwan, China) [35]. The life history characteristics included the developmental time of each stage; APOP, adult pre-oviposition period; oviposition days; mean fecundity of reproductive female; *Sxj* (*x* = age, *j* = stage), age-stage survival rate; *l_x_*, age-specific survival rate; *m_x_*, age-specific fecundity; *R_0_*, net reproduction; *r*, intrinsic rate of increase; *λ*, finite rate; *T*, mean generation time. The mean and standard error of each life history characteristic and the significant differences among *L. sticticalis* fed on different host plants were calculated and compared using the paired bootstrap test in TWO-SEX-MSChart (Version 2023, Laboratory of Theoretical Ecology, National Chung Hsing University, Taichung, Taiwan, China) [36], with the exception of *Sxj*, *l_x_*, and *m_x_*. Differences were considered significant at a 95% confidence interval and a *P*-value < 0.05. The population dynamics projection of *L. stieticatis*, when fed on different host plants over the next 120 days, were analyzed using TIMING-MSChart software (Version 2023, Laboratory of Theoretical Ecology, National Chung Hsing University, Taichung, Taiwan, China) [37].

The significant differences of pupal weight and flight ability indexes, such as total flight duration, distance, and average velocity, among *L. sticticalis* fed on different host plants were compared using ANOVA, followed by Tukey’s HSD post hoc comparisons. The significant differences in flight ability indexes between females and males of *L. sticticalis* fed on same host group were compared using independent samples *t*-test. ANOVA and independent samples *t*-test were performed in SPSS (Version 25, International Business Machines Crop., Armonk, New York, USA).

## 3. Results

### 3.1. Development, Survivorship, and Fecundity of Loxostege Sticticalis Fed on Different Green Manure Crops

For the host group of *V. villosa*, all larvae of *L. stieticatis* died before reaching the fifth instar stage, resulting in only raw data on survival rates and developmental time from egg to the fourth instar stage being obtained (Table 1), and no information was obtained regarding other life history characteristics such as the pupal weight or flight ability of adults. Thus, the host group of *V. villosa* was excluded from the comparison of significant differences. Although the significant difference between group *V. villosa* and the other three host groups could not be analyzed, it is worth noting that the average duration for egg hatching, first to second instar larvae development, and third instar larvae development were all longer in group *V. villosa* (Table 1).

The developmental time for each stage and fecundity for reproductive females of *L. stieticatis* fed on different host plants are shown in Table 1. For each host group, a corresponding host plant was provided to *L. stieticatis* from the egg stage to the end of the fifth instar larvae. Interestingly, eggs from the same batch exhibited varying hatching timelines due to their placement on different host plants. Comparing the host groups *C. album*, *P. sativum*, and *V. sativa*, the average egg hatching duration of the group *C. album* was shorter than that of the group *P. sativum* (*p* < 0.05). The first to second instar larvae development of group *C. album* was shorter than those of the other two host groups (*p* < 0.05). The development rate of *L. stieticatis* during its third to fifth instar larvae stages and pupal stage was the fastest in group *C. album*, followed by group *P. sativum*, and the slowest in group *V. sativa* (all *p* < 0.05). Adult longevity, regardless of sex, was significantly longer in group *C. album*, and the shortest male longevity was shown in group *P. sativum* (all *p* < 0.05). For reproductive females, the oviposition days in group *C. album* was the longest (*p* < 0.05), and the mean fecundity in group *C. album* was significantly higher than those of the other two host groups (*p* < 0.05).

**Figure 1:**
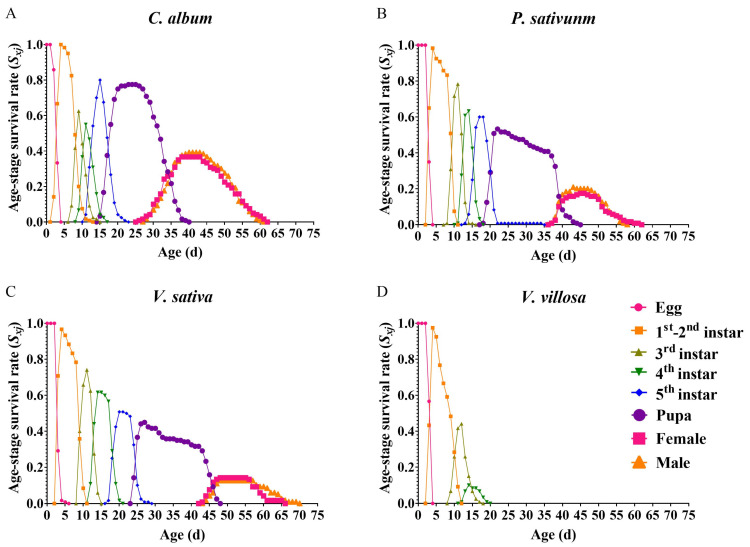
Age-stage specific survival rates (*s_xj_*) of *Loxostege sticticalis* fed on different host plants. (**A**) on *Chenopodium album*; (**B**) on *Pisum sativum*; (**C**) on *Vicia sativa*; (**D**) on *Vicia villosa*.

The age-stage survival rate curves (s_xj_) show the probability that a newborn will survive to age x and develop to stage j (Figure 1). The *s_xj_* curves show that 75.83% of the eggs of *L. stieticatis* in group *C. album* developed into adulthood (36.67% into females; 39.17% into males), but only 38.33% and 26.67% eggs of *L. stieticatis* in group *P. sativum* (17.50% into females; 20.83% into males) and *V. sativa* (14.17% into females; 12.50% into males), respectively (Figure 1A–C). However, all larvae of *L. stieticatis* died before reaching the fifth instar stage in group *V. villosa* (Figure 1D).

**Figure 2:**
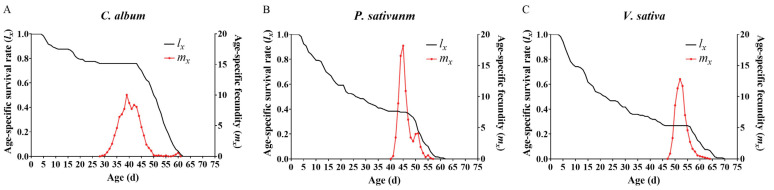
Age-specific survival rates (*l_x_*) and fecundities (*m_x_*) of *Loxostege sticticalis* fed on different host plants. (**A**) on *Chenopodium album*; (**B**) on *Pisum sativum*; (**C**) on *Vicia sativa*.

The age-specific survival rate (*l_x_*) and female adult age-specific fecundities (*m_x_*) are plotted in Figure 2. The *l_x_* curve describes the change in the survival rate of the group based on age, and the results showed that *L. stieticatis* in group *P. sativum* and *V. sativa* had a rapidly declining survivorship compared with that in group *C. album*. The *m_x_* curve describes the change in female mean fecundity of the group based on adult age, and the results showed that the reproduction of *L. stieticatis* began at ages 29 d, 40 d, and 48 d in groups *C. album*, *P. sativum*, and *V. sativa*, respectively. Although the maximal daily mean fecundity of female *L. stieticatis* in group *C. album* (at age 39 d, 10.03 eggs) was lower than those in group *P. sativum* (at age 45 d, 18.20 eggs) and *V. sativa* (at age 52 d, 12.81 eggs)*,* the egg reproduction duration in group *C. album* (27 days) was longer than those in the other two groups (both were 16 days).

### 3.2. Population Parameters of Loxostege Sticticalis Fed on Different Green Manure Crops

**Figure 3:**
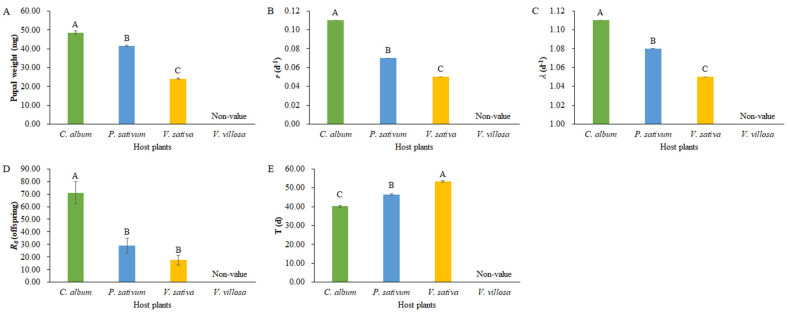
Pupal weight (**A**) and population life table parameters of *Loxostege sticticalis* fed on different host plants. (**B**) *r*, intrinsic rate of increase; (**C**) *λ*, finite rate of increase; (**D**) *R_0_*, net reproduction rate; (**E**) *T*, mean generation time. Capital letters indicate the significance of the difference.

The pupal weight of *L. sticticalis* fed on different host plants was significantly different, as follows: *C. album* > *P. sativum* > *V. sativa* (*F*_(2, 119)_ = 453.21, *p* < 0.01, Figure 3A).

The *r* and *λ* of *L. sticticalis* fed on different host plants were significantly different, as follows: *C. album* > *P. sativum* > *V. sativa* (all *p* < 0.05, Figure 3B,C). The *R_0_* of *L. sticticalis* in group *C. album* was 71.11 offspring per female, which is significantly higher than that in groups *P. sativum* (29.21 offspring per female) and *V. sativa* (29.21 offspring per female) (*p* < 0.05, Figure 3D). In addition, the *T* of *L. sticticalis* in group *C. album* (40.16 d) was the shortest, followed by group *P. sativum* (46.30 d), while that of group *V. sativa* (53.27 d) was the longest (*p* < 0.05, Figure 3E).

### 3.3. Population Projection of Loxostege Sticticalis Fed on Different Green Manure Crops

**Figure 4:**
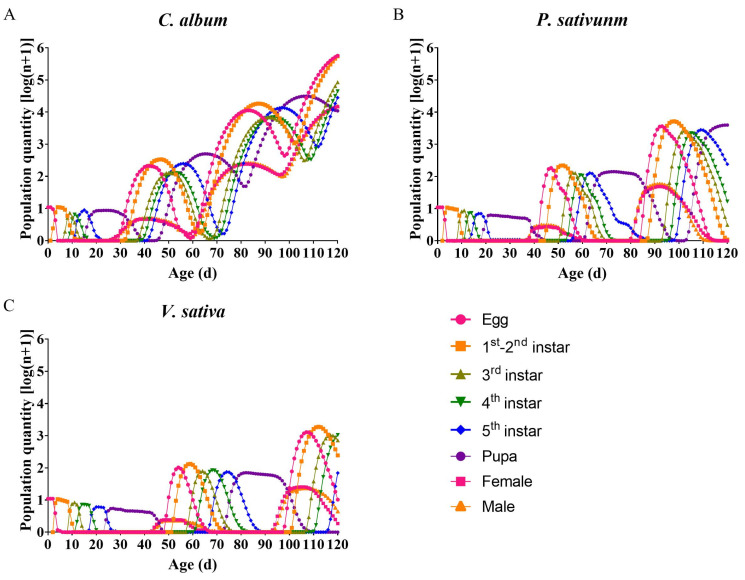
Predicted population dynamics of *Loxostege sticticalis* over 120 days based on life table data when fed on different host plants. (**A**) on *Chenopodium album*; (**B**) on *Pisum sativum*; (**C**) on *Vicia sativa*.

The population dynamics predicted by TIMING-MSChart (Version 2023, Laboratory of Theoretical Ecology, National Chung Hsing University, Taichung, Taiwan, China) for the next 120 days, based on *L. sticticalis* from 10 eggs feeding on different host plants, are illustrated in Figure 4. When feeding on the suitable host *C. album*, the predicted population steadily and rapidly increased, reaching the binge-feeding stage of fourth-generation larvae within 120 days with the larval count increasing by six orders of magnitude (Figure 4A). However, when feeding on the hosts *P. sativunm* and *V. sativa*, the predicted population growth was slower, and at 120 days, the populations were in the third-generation pupal stage and the third-generation larval stage, respectively, and the third-generation larva number increased by 3–4 orders of magnitude in both host groups (Figure 4B,C).

### 3.4. Flight Ability of Loxostege Sticticalis after Feeding on Different Green Manure Crops

**Figure 5:**
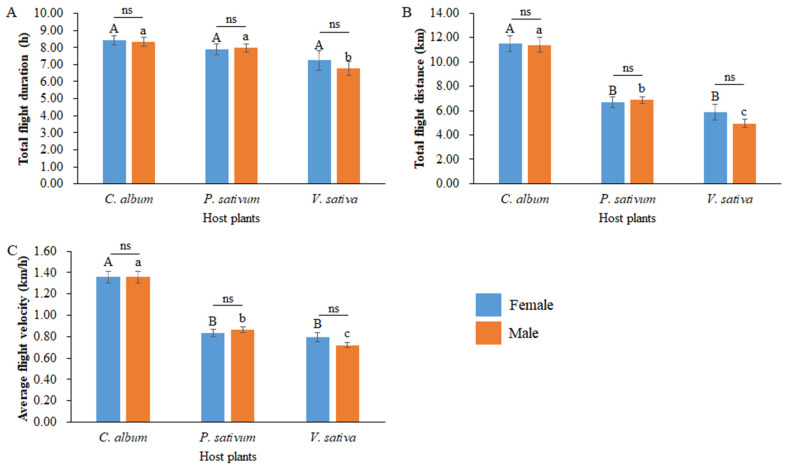
Flight ability parameters of *Loxostege sticticalis* fed on different host plants. (**A**) Total flight duration (h); (**B**) total flight distance (km); (**C**) average flight velocity (km/h). Capital letters indicate the significant differences in flight ability indexes among female *L. sticticalis* fed on different host plants. Lowercase letters indicate the significant differences in flight ability indexes among male *L. sticticalis* fed on different host plants. The significant differences in flight ability indexes between females and males of *L. sticticalis* fed on the same host group are represented by ‘ns’, which indicate no significant difference.

Flight ability data of adult *L. sticticalis* from different host plants were examined by gender. For each host group, there were no significant differences between females and males in terms of total flight duration (*C. album*: *t* = 0.273, df = 46, *p* = 0.786; *P. sativum*: *t* = −0.211, df = 46, *p* = 0.834; *V. sativa*: *t* = 0.646, df = 46, *p* = 0.522; Figure 5A), total flight distance (*C. album*: *t* = 0.132, df = 46, *p* = 0.895; *P. sativum*: *t* = −0.372, df = 38.051, *p* = 0.712; *V. sativa*: *t* = 1.268, df = 36.538, *p* = 0.213; Figure 5B), or average flight velocity (*C. album*: *t* = −0.015, df = 46, *p* = 0.988; *P. sativum*: *t* = −0.706, df = 46, *p* = 0.483; *V. sativa*: *t* = 1.535, df = 36.047, *p* = 0.134; Figure 5C). For females, feeding on different hosts had a negligible impact on total flight duration (*F*_(2, 71)_ = 2.111, *p* = 0.129, Figure 5A). However, when feeding on *P. sativum* and *V. sativa*, there was a significant reduction in the total flight distance (*F*_(2, 71)_ = 27.250, *p* < 0.01, Figure 5B) and average flight speed (*F*_(2, 71)_ = 49.305, *p* < 0.01, Figure 5C). For males, feeding on *V. sativa* resulted in a decrease in the total flight duration (*F*_(2, 71)_ = 7.075, *p* = 0.002, Figure 5A), while feeding on *P. sativum* and *V. sativa* also caused a significant reduction in both the total flight distance (*F*_(2, 71)_ = 57.836, *p* < 0.01, Figure 5B) and average flight velocity (*F*_(2, 71)_ = 80.489, *p* < 0.01, Figure 5C).

## 4. Discussion

The host range of *L. sticticalis* is broad, and it prefers to feed on plants from the families Compositae, Leguminosae, Chenopodiaceae, Polygonaceae, and Brassicaceae, among others [20]. However, the performance of *L. sticticalis* differs significantly depending on the host plant it consumes. For example, compared to *C. album*, *L. sticticalis* experienced a longer development time, lower pupal weight, and reduced fecundity and mating success rate when feeding on its host plants maize and potatoes [38]. The larval survival rate, life table parameters (*R_0_*, *r*, and *λ*), and fecundity of *L. sticticalis* reared on *Helianthus annuus* L., *Triticum aestivum* L., and *Glycine max* (L.) Merr. were all lower than in those reared on *C. album* [39]. In our study, *L. sticticalis* also exhibited the highest performance on its suitable host *C. album*. Compared to the three other green manure crops, feeding on *C. album* resulted in the highest survival rate for *L. sticticalis* larvae, a shorter development duration for both larvae and pupae, a heavier pupal weight, a longer adult longevity and great number of oviposition days, a higher mean fecundity of reproductive females, and the highest net reproduction rate (*R_0_*), intrinsic rate of increase (*r*), and finite rate of increase (*λ*). The mean generation time (*T*) was also the shortest. Notably, *L. sticticalis* feeding on *V. villosa* failed to complete their generation development, with a 100% mortality rate during the larval stage. Therefore, *V. villosa* is not a host plant for *L. sticticalis*, and increasing the planting proportion of *V. villosa* as green manure crops in the ‘Three North’ regions of China will not increase the risk of *L. sticticalis* outbreaks.

Some green manure crops may feed and provide a habitat or refuge for migratory lepidopteran pests, potentially leading to increased pest populations. *Spodoptera litura* (F.) can successfully survive and reproduce using three green manure crops—sesbania, sunn hemp, and rapeseed—which are widely planted in Taiwan [16]. The fall armyworm, *Spodoptera frugiperda* (Smith), can complete generation development when feeding on *V. villosa* and *V. sativa*, although its mean generation time is significantly longer and its egg production is significantly reduced compared with feeding on suitable host maize [30]. In our study, by using the age-stage two-sex life table, regardless of the developmental time and fecundity for reproductive females, i.e., oviposition days, and mean fecundity, or the population parameters, i.e., the net reproductive rate (*R_0_*), intrinsic rate of increase (*r*), finite rate of increase (*λ*), and the mean generation time (*T*), all of our results demonstrated that *L. sticticalis* was well-adapted to these two green manure crops (*P. sativam* and *V. sativa*). Furthermore, population projections predicted the periodic emergence of adults when *L. sticticalis* fed on these two green manure crops. However, the host adaptability of *L. sticticalis* to these two green manure crops was significantly lower than that to *C. album*.

The *L. sticticalis* has always been one of the primary migratory crop pests in the ‘Three North’ regions of China [40,41]. Migration is a behavioral strategy that has evolved over time in *L. sticticalis*, and it is the primary cause of frequent outbreaks and significant crop yield and economic losses [17,42]. The migration of a large number of adults often leads to a significant increase in larval populations at the site of migration [43,44]. There also exists an interactive relationship between the migration patterns of *L. sticticalis* and their reproductive behavior [45]. The strength of flight ability is related to the migration path and destination of *L. sticticalis*. In our study, *L. sticticalis* was able to adapt to these two green manure crops (*P. sativam* and *V. sativa*), but its flight ability, particularly the total flight distance, was significantly reduced compared to when feeding on *C. album*. When the feeding environment conditions, supplemental nutrition and adult age were consistent [34], the variation in the flight ability of *L. sticticalis* was primarily attributed to differences in nutrients and insect-resistant compounds acquired from their host plants during the larval stage. That is to say, the promotion and cultivation of specific green manure varieties can partially impede the migration capacity of *L. sticticalis*. Our research findings will serve as an experimental basis for the promotion and cultivation of green manure crops in the source areas, diapause zones, and migration paths of *L. sticticalis*.

Different host plants exert a significant influence on the performance of herbivorous insects, affecting their preferences, growth and development, reproduction, and other aspects [30,38,46,47]. The observed effects are commonly attributed to variations in the nutrient content, secondary substances, and unique compounds present in different host plants [48,49]. In a meta-analysis of the effects of within-population plant trait variance on herbivore performance using 457 performance datasets from 53 species of insect herbivores, Wetzel et al. [50] showed that plants contribute to the suppression of herbivore populations through variations in nutrient levels. The secondary plant metabolite Cucurbitacin B and various flavonoids caused a negative impact on the growth and development of the melon aphid (*Aphis gossypii*) and the legume pod borer (*Helicoverpa armigera*), respectively [51,52]. The comprehensive impact of different green manure crops on *L. sticticalis* was observed in this experiment. In terms of growth, development, reproduction, and flight ability, *L. sticticalis* exhibited the highest performance on its suitable host *C. album*, followed by *P. sativam* and *V. sativa*; however, it was not able to adapt to *V. villosa*. Interestingly, the presence of green manure crops in close proximity to the eggs has a significant impact on their hatching time. The results of this study demonstrate that when eggs from the same batch of *L. sticticalis* were placed in the same container as *V. villosa*, they exhibited the longest hatching period. This was followed by *P. sativam* and *V. sativa*, while placing them in the same container as *C. album* resulted in the shortest hatching period for these eggs (Figure 1). This also suggests that plant volatile compounds can not only influence the selection of migratory pests for oviposition [53,54,55], but can also directly impact egg hatching. Further research is needed to identify the key compounds present in different host plants that affect the growth, development, reproduction, and flight ability of *L. sticticalis*.

## 5. Conclusions

A comprehensive evaluation of the effects of three major legume green manure crops in China (*P. sativam*, *V. sativa*, and *V. villosa*) on the performance of *L. sticticalis* was carried out using a two-sex life table and flight mill test approach. Our findings suggest that *L. sticticalis* cannot complete generational development with *V. villosa*, while it exhibits reduced performance on *P. sativam* and *V. sativa* compared to the suitable host *C. album*, due to prolonged egg and larval development periods, shortened adult stage, decreased fecundity, and impaired flight capacity. However, both the population parameters (*R_0_*, *r*, *λ*, and *T*) and the population prediction results indicate that *L. sticticalis* is capable of adapting to *P. sativam* and *V. sativa*. Our research provides a foundation for selecting appropriate green manure varieties and implementing effective pest control measures during their cultivation.

## Figures and Tables

**Table 1:** Developmental time for each stage and fecundity for reproductive females of *Loxostege sticticalis* fed on different host plants.

Indexes	Host Plants							
	*C. album*		*P. sativum*		*V. sativa*		*V. villosa*	
	*n*	Mean ± SE	*n*	Mean ± SE	*n*	Mean ± SE	*n*	Mean ± SE
Developmental time of each stage (d)								
Egg	120	3.19 ± 0.06 b	120	3.35 ± 0.04 a	120	3.32 ± 0.05 ab	120	3.57 ± 0.05
first to second instar	107	5.58 ± 0.09 b	95	6.37 ± 0.05 a	89	6.32 ± 0.05 a	64	7.08 ± 0.10
third instar	105	2.22 ± 0.04 c	84	3.02 ± 0.02 b	86	3.74 ± 0.05 a	24	3.96 ± 0.15
fourth instar	105	2.27 ± 0.05 c	74	2.96 ± 0.06 b	62	5.32 ± 0.07 a	-	-
fifth instar	96	4.48 ± 0.08 c	66	4.72 ± 0.08 b	56	6.41 ± 0.08 a	-	-
Pupa	91	15.48 ± 0.26 c	46	19.63 ± 0.21 b	32	20.91 ± 0.21 a	-	-
Adult							-	-
Female	44	19.93 ± 0.65 a	21	13.38 ± 0.60 b	17	14.17 ± 0.50 b	-	-
Male	47	18.98 ± 0.38 a	25	12.92 ± 0.58 c	15	16.60 ± 0.79 b	-	-
Fecundity (for reproductive female)								
APOP (d)	44	4.13 ± 0.15 a	21	3.57 ± 0.15 b	17	4.65 ± 0.27 a	-	-
Oviposition days	44	10.25 ± 0.25 a	21	7.28 ± 0.26 b	17	7.88 ± 0.24 b	-	-
Mean fecundity	44	194.03 ± 5.11 a	21	166.90 ± 6.90 b	17	123.96 ± 3.98 c	-	-

Lowercase letters indicate the significance of the difference (compared using the paired bootstrap test, *p*-value < 0.05).

## Data Availability

The data presented in this study are available on request from the corresponding author.
